# Elucidation of White Matter Tracts of the Human Amygdala by Detailed Comparison between High-Resolution Postmortem Magnetic Resonance Imaging and Histology

**DOI:** 10.3389/fnana.2017.00016

**Published:** 2017-03-14

**Authors:** Susumu Mori, Yusuke Kageyama, Zhipeng Hou, Manisha Aggarwal, Jaymin Patel, Timothy Brown, Michael I. Miller, Dan Wu, Juan C. Troncoso

**Affiliations:** ^1^Russell H. Morgan Department of Radiology and Radiological Science, Johns Hopkins University School of MedicineBaltimore, MD, USA; ^2^Department of Pathology, Division of Neuropathology, Johns Hopkins University School of MedicineBaltimore, MD, USA; ^3^Center for Imaging Science, Johns Hopkins UniversityBaltimore, MD, USA; ^4^Institute for Computational Medicine, Johns Hopkins UniversityBaltimore, MD, USA; ^5^Department of Biomedical Engineering, Johns Hopkins University School of MedicineBaltimore, MD, USA

**Keywords:** white matter anatomy, amygdala, MRI, diffusion tensor imaging, microimaging, histology, stria terminalis, amygdalofugal pathway

## Abstract

The amygdala has attracted considerable research interest because of its potential involvement in various neuropsychiatric disorders. Recently, attempts have been made using magnetic resonance imaging (MRI) to evaluate the integrity of the axonal connections to and from the amygdala under pathological conditions. Although amygdalar pathways have been studied extensively in animal models, anatomical references for the human brain are limited to histology-based resources from a small number of slice locations, orientations and annotations. In the present study, we performed high-resolution (250 μm) MRI of postmortem human brains followed by serial histology sectioning. The histology data were used to identify amygdalar pathways, and the anatomical delineation of the assigned structures was extended into 3D using the MRI data. We were able to define the detailed anatomy of the stria terminalis and amygdalofugal pathway, as well as the anatomy of the nearby basal forebrain areas, including the substantia innominata. The present results will help us understand in detail the white matter structures associated with the amygdala, and will serve as an anatomical reference for the design of *in vivo* MRI studies and interpretation of their data.

## Introduction

It is widely accepted that the amygdala is involved in various emotional responses (reviewed by Amaral, 2003; Phelps and LeDoux, 2005), in which white matter connectivity plays an essential role. Amygdalar connectivity has been studied extensively using animal models, which have shown that the stria terminalis and amygdalofugal pathways serve two major connections of the amygdala. The stria terminalis is an efferent pathway that connects the amygdala to the septal area of the basal forebrain and hypothalamus. It is a poorly myelinated tract, 2–4 mm in diameter, which has only partially been identified by *in vivo* diffusion tensor imaging (DTI) or magnetic resonance imaging (MRI) of the human brain. Despite its name, the amygdalofugal pathway is believed to contain both afferent and efferent connections to the basal forebrain, hypothalamus and thalamus. Its location has been described using histology sections (Mai et al., 2008; Yilmazer-Hanke, 2012) and drawings of the human brain (Nieuwenhuys et al., 1988), which indicate a projection (approximately 1 mm in diameter) to the basal forebrain. Animal studies revealed that the amygdala has extensive connections to various cortical areas and the brainstem, but the exact locations of these axonal tracts have not been well defined in the human brain, probably because they do not form discrete and independent bundles.

Anatomical MRI has been used to study the role of the amygdala in various pathologies, including major depression (Munn et al., 2007; Paparrigopoulos et al., 2008), schizophrenia (Wright et al., 2000) and autism (Amaral et al., 2003). A comprehensive meta-analysis by Brierley et al. (2002) identified 39 volumetric MRI studies of the amygdala, indicating a high level of interest as well as highlighting the difficulties of studying this complex structure. For MRI-based anatomical analysis of the amygdala, T1-weighted images have been used most frequently and anatomical protocols for structural delineation have been proposed (Watson et al., 1992; Achten et al., 1998; Makris et al., 1999; Pruessner et al., 2000; Bonilha et al., 2004; Entis et al., 2012). In recent years, *in vivo* DTI has also been used in an attempt to delineate white matter tracts related to the amygdala (Wakana et al., 2004; Mori et al., 2005, 2008; Kamali et al., 2015, 2016) or its connectivity (Solano-Castiella et al., 2010, 2011; Bach et al., 2011; Saygin et al., 2011; Kamali et al., 2015, 2016; Rafal et al., 2015). However, these small white matter tracts are intricately linked with the target structures, requiring high-resolution observation that is often beyond the resolution achievable in *in vivo* imaging. As high angular resolution diffusion imaging and probabilistic tractography technologies evolve, sub-voxel connectivity analysis based on low-resolution *in vivo* diffusion MRI is becoming available for the study of the complex patterns of amygdalar connections (e.g., Saygin et al., 2011; Ford and Kensinger, 2014; Wiech et al., 2014; Bonilha et al., 2015; Fani et al., 2015; Souza-Queiroz et al., 2016). The ability of postmortem MRI and histology to reveal the microscopic anatomy of these pathways is a precious resource as anatomical guidance.

In the present study, we used three postmortem human brain samples to acquire 3D MRI data from white matter structures related to the human amygdala at the mesoscale (250 μm resolution). One of the samples was then serially sectioned to create a standard for anatomical assignment. This dataset provided us with several unique opportunities to deepen our understanding of the white matter anatomy of the amygdala. We were especially interested in delineating the anatomy of the basal forebrain area directly rostromedial to the amygdala, often called the substantia innominata (meaning “unnamed substance”), which has consistently shown lateral structural alignment in past DTI studies and is known to contain several nuclei and small white matter tracts, including the amygdalofugal pathway. Another area of interest included the white matter at the ventral portion of the stria terminalis, located at the caudodorsal end of the amygdala and only partially assigned by conventional DTI to date. Here, we describe the anatomy of these important areas using serial histology sections to provide anatomical landmarks and clarify the MRI contrast of the white matter associated with amygdalar pathways. In addition, we will discuss the unique challenges we encountered with the present preliminary mesoscale anatomical studies for consideration in future population-based studies.

## Materials and Methods

### Human Brain Tissue

The study was based on three postmortem brains tissues which became available after routine pathology services (one male, 22 years; two females, 16 and 21 years) from the Brain Resource Center, Department of Pathology, Johns Hopkins University. The study was conducted under a protocol approved by the Institutional Review Board (IRB) of Johns Hopkins University, School of Medicine. The usage of these de-identified tissues for the research purpose was approved by the IRB. None of the subjects had any known neurological condition. The brain tissues were fixed in 10% formaldehyde (Hydrol Chemical Company, Yeadon, PA, USA) for more than 2 weeks, then sectioned into 10–15 mm-thick coronal slabs. Tissue blocks approximately 40 × 20 mm in area were cut from the slabs that contained the amygdala.

### Histology

Following the postmortem MRI, one of the tissue specimens (male, 22 years) was processed for histological analysis. The specimen was embedded in paraffin blocks, cut into 10 μm-thick sections at 200 μm intervals, and stained using Luxol fast blue with hematoxylin and eosin. Deparaffinized sections were mounted and incubated in Luxol fast blue solution for 20 min at 60°C, and the slides were washed thoroughly with distilled water. The sections were then washed with a 0.05% lithium carbonate solution for 5 s and 70% alcohol for 10 s, twice, to differentiate the myelin structure. The slides were washed thoroughly again with distilled water. The sections were then stained with hematoxylin 7211 (Thermo Fisher Scientific, Waltham, MA, USA) for a few seconds, and washed thoroughly with warm tap water. Finally, the sections were immersed in eosin Y solution (Thermo Fisher Scientific, Waltham, MA, USA) for 1 min, rinsed in distilled water, and dehydrated through 95% alcohol and xylene. Images were captured under a Zeiss Axio Observer.Z1 microscope equipped with an AxioCam MRc camera (Carl Zeiss Microscopy, Thornwood, NY, USA) and a ×5 objective.

### MRI Scans

The MRI sequence was based on a 3D multiple echo sequence (Mori and van Zijl, 1998; Xue et al., 2001) with four echoes acquired for each excitation. Diffusion-weighted images were acquired with a field of view of typically 40 × 30 × 16 mm and an imaging matrix of 160 × 120 × 64, which was zero-filled to 320 × 240 × 128 after the spectral data were apodized using a 10% trapezoidal function. The native pixel size was 250 μm isotropic. Eight diffusion-weighted images were acquired with different diffusion gradient directions, with *b*-values in the 1200–1700 s/mm^2^ range. For diffusion-weighted images, a repetition time of 0.9 s, echo time of 37 ms, and two signal averages were used, for a total imaging time of 24 h.

### Tensor Calculation and Tract Reconstruction

Diffusion MRI data processing was based on conventional tensor calculation using DTIStudio (Jiang et al., 2006). Images presented in this article included b0 images (least diffusion-weighted images) and trace images (the sums of the diagonal elements of the diffusion tensor). In addition, color-coded orientation maps were created by combining the principal eigenvectors and Westin’s linear (CL) index (Westin et al., 2002). For color-coded orientation maps, fractional anisotropy (FA) is widely used, but for high-resolution imaging, in which partial voluming of structures with different alignment orientations within a voxel occurs less frequently, the entire tissue tends to have relatively higher FA values, leading to a lack of contrast. Westin’s CL index has high values with linear (cigar-shaped) anisotropy and suppresses high-FA regions with planar (disk-shaped) anisotropy, thus providing higher anatomical contrast. For tract reconstruction, we used the fiber assignment by continuous tracking (FACT) deterministic algorithm implemented in DTIStudio. This is an operator-driven reconstruction tool and is used to help viewers understand the 3D architecture of white matter tracts.

### Structural Assignment and Segmentation

Assignment of the white matter structures and amygdalar nuclei was based on the comparison between our histology sections and the panels in the atlases of Mai et al. (2008) and Yilmazer-Hanke (2012). However, these assignments were limited to the 2D histology panels available in these atlases. The availability of the 3D mesoscale images and confirmation of the continuity of white matter tracts by 3D tractography greatly enhanced the validity of the assignments. The manual segmentation of amygdalar nuclei was performed by TB and SM, where the laminar structures that were visible in the mesoscale image were important clues. These nucleus segmentations improved our understanding of the relationships between the amygdala and associated white matter tracts.

## Results

### Slice-by-Slice Comparison of High-Resolution MRI and Histology Sections

**Figure 1:**
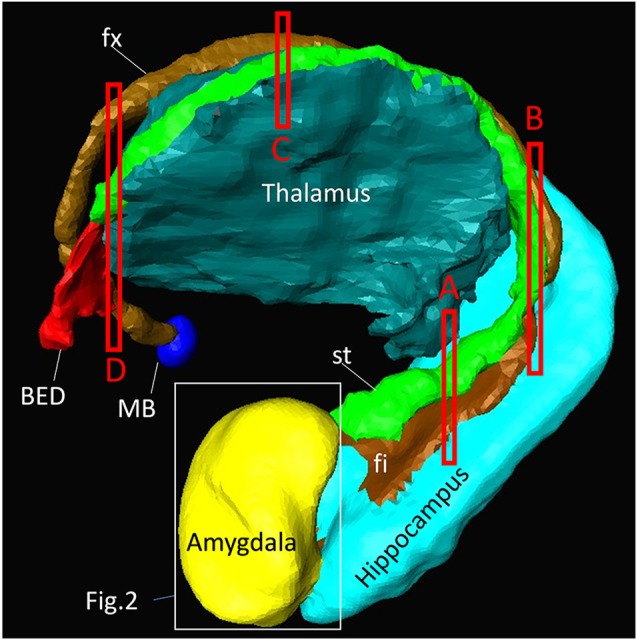
**Overview of the anatomy of the amygdala and its relationships with other nearby brain structures.** Segmentation of these structures was based on high-resolution *in vivo* magnetization-prepared rapid gradient-echo (MP-RAGE) data at 0.6 mm resolution. One of the major afferent/efferent pathways of the amygdala is the stria terminalis (st) and the major destination is the bed nucleus (BED). The fimbria (fi) and fornix (fx) are hippocampal pathways with similar C-shaped trajectories, and are often difficult to resolve from the stria terminalis in lower-resolution images. The white bounding box shows the approximate areas described in Figure 2. The red bounding boxes (labeled **A–D**) indicate the slice locations in Figure 4. The other major pathway of the amygdala, the amygdalofugal pathway, could not be identified on the MP-RAGE image. MB, mammillary body.

Figure 1 shows a macroscopic view of the anatomy of long-range connections and gray matter structures of the amygdala, reconstructed from previously available *in vivo* T1-weighted and DTI data^1^. This served as an anatomical reference on a whole-brain scale. The amygdala was located at the rostral face of the hippocampus and stria terminalis (st), one of the major afferent/efferent pathways of the amygdala. In the temporal lobe, the stria terminalis traveled along the fimbria (fi) and fornix (fx), which were connected to the hippocampus. Although these tracts were adjacent in the 3D space, the location of the stria terminalis was anatomically very distinct from the fimbria and fornix as they traveled along the opposite bank of the temporal horn of the lateral ventricle. The stria terminalis and fornix had similar C-shape trajectories around the thalamus and reached the basal forebrain and hypothalamus. One of the major destinations of the stria terminalis was the bed nucleus (BED), which was located close to the amygdala, completing the large loop. The approximate locations of the high-resolution data in subsequent sections are also indicated in Figure 1.

**Figure 2:**
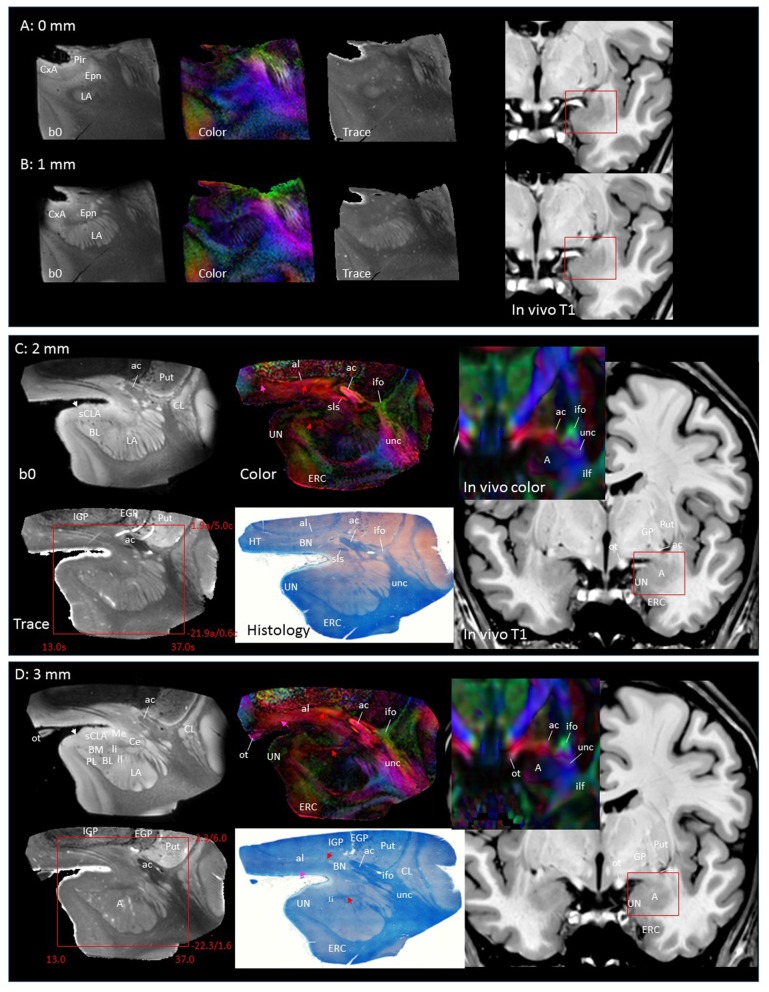

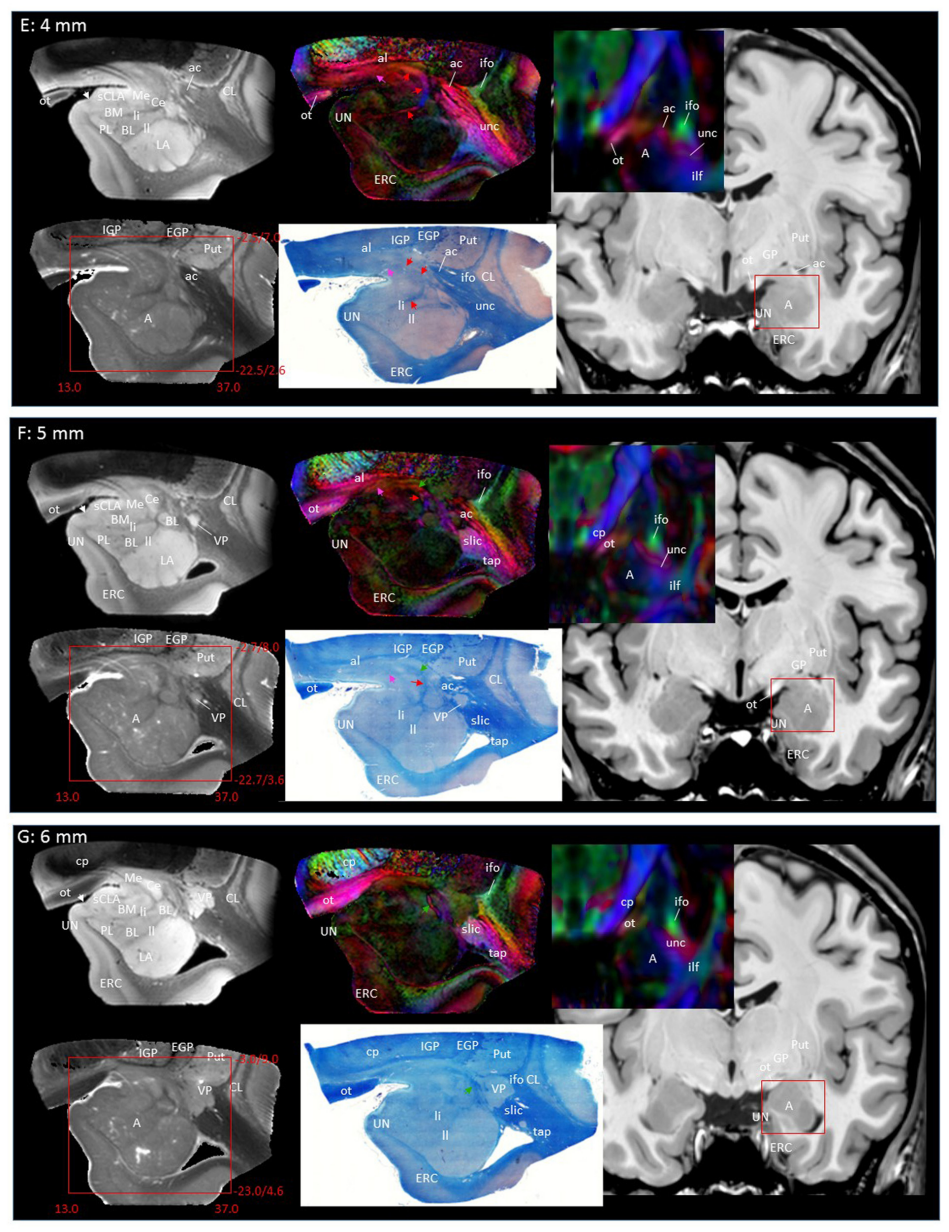

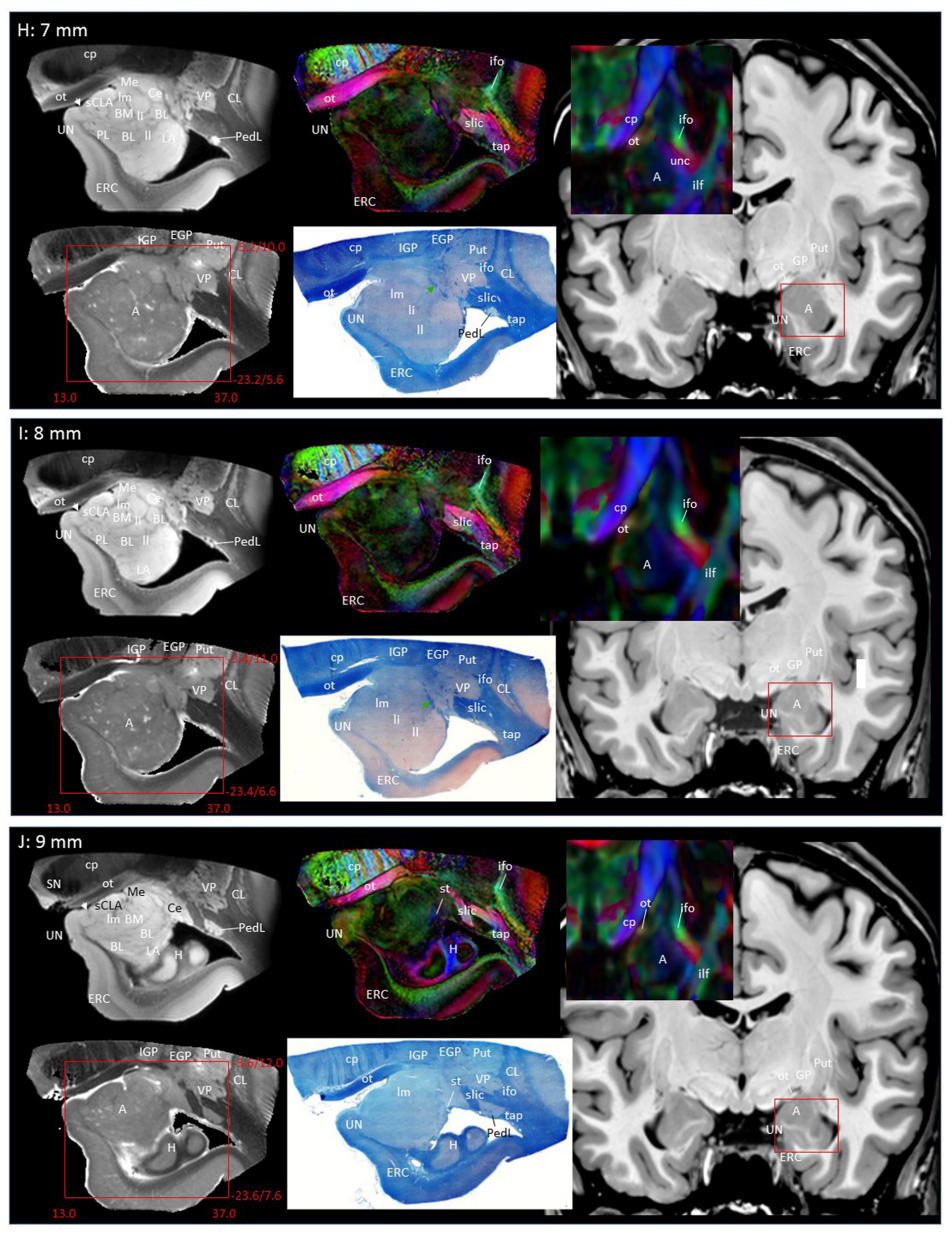

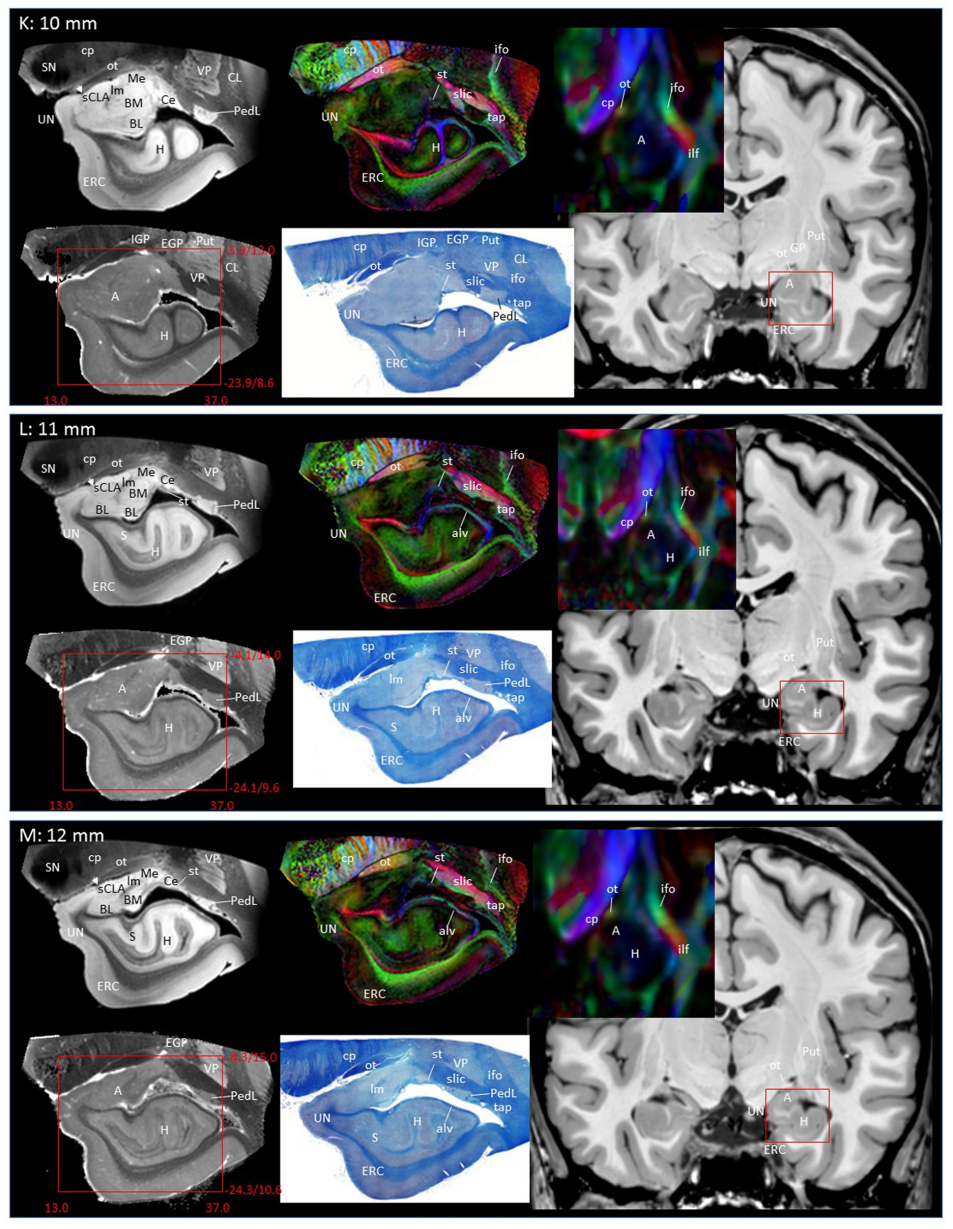

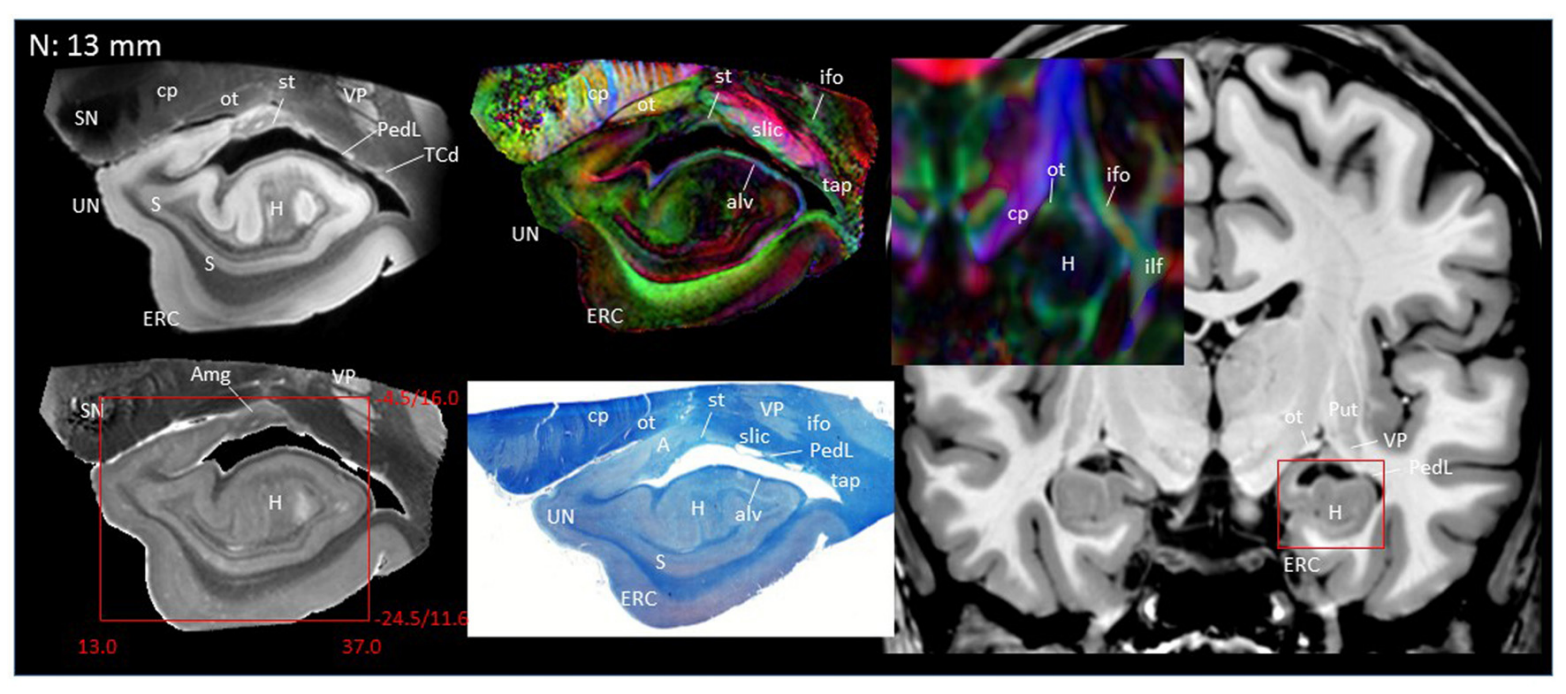
**Coronal panels for detailed anatomical delineation of the amygdala using postmortem diffusion tensor imaging (DTI) and histology (Luxol fast blue with hematoxylin–eosin staining), compared with an example of *in vivo* T1 and DTI images.** From the postmortem DTI data, b0, trace and color-coded orientation maps are shown at 14 coronal slice levels. Panels **(A,B)** were from a different postmortem coronal slab than **(C–N)**. Panel **(A)** corresponds to the rostral end of the amygdala that defines 0 mm to indicate the slice separation of the subsequent panels, and panel **(N)** shows the caudal end (13 mm). The red bounding boxes represent a 24 × 20 mm field of view at the corresponding locations on the *in vivo* and postmortem images with the MNI coordinates (s, sagittal; a, axial; c, coronal). Colored arrows indicate the locations of the reconstructed white matter structures shown in Figure 3. White arrows indicate the semiannular sulcus (amygdaloid fissure) defining the medial boundary of the amygdala. Abbreviations: A, amygdala; BL, basolateral amygdaloid nucleus; BN, basal nucleus diffuse part; BM, basomedial nucleus; Ce, central nucleus; CL, claustrum; CxA, amygdalocortical transition area; EGP, external globus pallidus; Epn, endopiriform nucleus; ERC, entorhinal cortex; GP, globus pallidus; H, hippocampus; HT, hypothalamus; IGP, internal globus pallidus; LA, lateral nucleus; Me, medial nucleus; PedL, peduncle of the lentiform nucleus; Pir, piriform cortex; PL, paralaminar nucleus; Put, putamen; S, subiculum; sCLA, superficial cortex-like amygdala; SN, substantia nigra; TCd, tail of caudate; UN, uncus; VP, ventral putamen; ac, anterior commissure; al, ansa lenticularis; alv, alveus; cp, cerebral peduncle; ifo, inferior fronto-occipital fasciculus; ilf, inferior longitudinal fasciculus; li, intermediate medullary lamina; ll, lateral medullary lamina; lm, medial medullary lamina; ot, optic tract; slic, sublenticular part of the internal capsule; sls, sublenticular stria; st, stria terminalis; tap, tapetum; unc, uncinated fasciculus.

**Figure 3:**
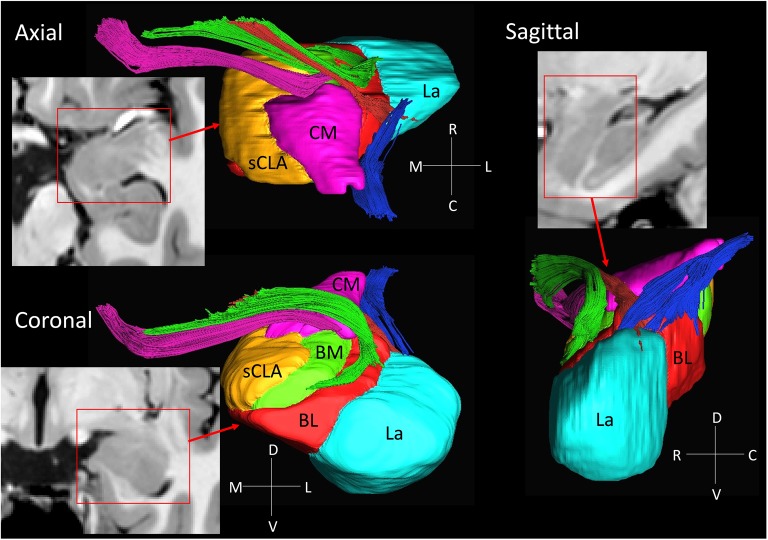
**3D reconstruction of amygdalar nuclei and associated white matter tracts.** From the rostral surface, several bundles of amygdalofugal pathways could be constructed, which innervate the medullary lamina between the nuclei. The locations of these bundles in the coronal sections are indicated by the arrows in Figure 2, using the same color scheme as the reconstructed bundles (pink, green and red). The stria terminalis (blue) reaches the amygdala from the caudal face, which connects to the centromedial nuclei, while several branches extend to the basolateral and lateral nuclei. Abbreviations: BL, basolateral (red); BM, basomedial (green); CM, centromedial (pink); La, lateral (light blue); sCLA, superficial cortex-like amygdala (yellow).

In Figure 2, slice-by-slice comparisons of postmortem MRI, histology, and previous *in vivo* MRI are shown, as well as detailed structural assignments. The postmortem MRI and histology data in Figure 2 are from the same brain. In the postmortem MRI, intra-amygdalar anatomical contrasts were obtained from the high myelin content of the laminar structures (lm, li, ll; medial, intermediate and lateral medullary lamina, respectively), which appeared darker on the b0 (T2-weighted) images, demarcating the boundaries of several nuclei, although the boundary of the medial (Me) and central (Ce) nuclei was obscure at the rostral end. The boundary between the entorhinal cortex (ERC) and superficial cortex-like amygdala (sCLA) was clearly defined by the semiannular sulcus (sas, white arrows), although the boundary between the sCLA and basomedial (BM) and medial nuclei was not always clear in the rostral area. For the white matter tracts, comparison of the histology sections and 3D trajectory information provided by tractography (Figure 3) greatly facilitated the structural assignment.

Figures 2A,B covers the first 1 mm of the amygdala from the rostral end, where the transition from the piriform cortex (Pir) to the amygdalocortical (CxA) occurred. The highly fibrous lateral amygdala nuclei (LA) also started at this level. The body of the amygdala and associated white matter tracts can be seen in the subsequent coronal slices at 2–13 mm (Figures 2C–N). At the 2 mm level (Figure 2C), the basolateral nuclei (BL) and the cortex-like amygdala (sCLA) appeared at the 2 mm level (Figure 2C). The area inferior to the globus pallidus (GP), labeled as the basal nucleus (BN), is known to contain both diffuse gray matter nuclei and white matter tracts, and demonstrates strong structural alignment between the amygdala and hypothalamus (HT). This area contained several white matter tracts, including the anterior commissure (ac) and ansa lenticularis (al), and the tractography results confirmed the extensions of the amygdalofugal pathway in this area (arrows). The area that contains these structures is often collectively called the substantia innominata.

Through the 2–7 mm levels (Figures 2C–H), multiple basal nuclei (LA, lateral; BM, basomedial and BL, basolateral) can be recognized, demarcated by the laminar structures. The mesoscale images and histology sections captured how bundles of the amygdalofugal pathways exited the amygdala and projected toward the basal forebrain and hypothalamus. Throughout these areas, a limited number of white matter tracts could be clearly identified in the *in vivo* images, including the anterior commissure (ac), optic tract (ot) and the inferior fronto-occipital fasciculus (ifo). As also shown in Figure 3, the amygdalofugal pathways had strong associations with the central and basolateral nuclei, in agreement with previous animal studies (Nieuwenhuys et al., 1988). As the sections enter the posterior part of the amygdala (8–13 mm, Figures 2I–N), the location of the stria terminalis becomes recognizable and its close relationships with the main output structures, as well as the medial and basal nuclei, can be appreciated.

### 3D Reconstruction of Nuclei and White Matter Tracts of the Amygdala

Figure 3 shows 3D reconstructions of multiple nuclei of the amygdala and associated white matter tracts. The defined nuclei were the (sCLA, yellow), the centromedial (Me/Ce, pink), (BM, green), basolateral (BL, red) and lateral (La, light blue) nuclei. The white matter tracts that could be clearly identified in both postmortem MRI and corresponding histology sections were reconstructed in 3D using deterministic tractography. The locations of these white matter tracts in the 2D presentations are also shown in Figure 2. The stria terminalis (Figures 2J–N, 3, blue) entered the amygdala from the caudal end and projected along the lateral surface of the centromedial nucleus, continuing toward the basolateral and lateral nuclei. Part of the projection entered into the lateral medullary lamina (Figures 2D–I, ll). From the rostral side, several bundles of large pathways left the amygdala and entered into the basal forebrain areas with convoluted trajectories. In previous reports based on histology, these fibers had been collectively called the amygdalofugal pathway. This tract was also recently revealed using *in vivo* DTI (Kamali et al., 2016). Several bundles could be differentiated as they entered the amygdala, forming clearly identifiable branches (Figures 2C–I, colored arrows).

**Figure 4:**
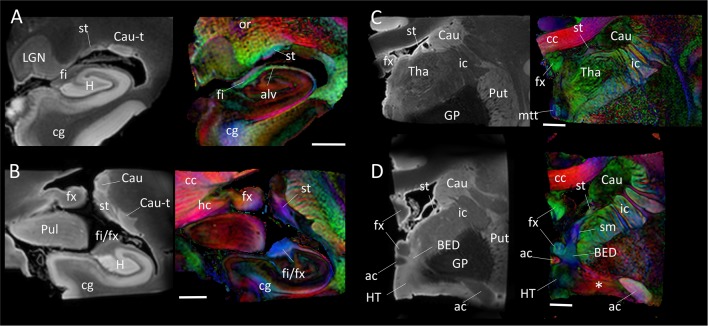
**Trajectory of the stria terminalis.** Panel (**A–D**) show high-resolution MR images at four different locations indicated in Figure 1. The stria terminalis (st) and the fornix (fx) have spatially similar, but anatomically distinct, trajectories. For the entire trajectory, the stria terminalis remains in close proximity to the caudate. Compared with the fornix, the poorly myelinated stria terminalis has lower fractional anisotropy (FA) value and higher T2 intensity, more similar to that of gray matter structures. Scale bars, 5 mm. Abbreviations are the same as in Figure 2, with the addition of: Cau, caudate; Cau-t, tail of caudate; HT, hypothalamus; LGN, lateral geniculate nucleus; Pul, pulvinar; Tha, thalamus; cc, corpus callosum; cg, cingulate; ic, internal capsule; mtt, mammillothalamic tract; or, optic radiation; sm, stria medullaris.

Figure 4 shows the trajectory of the long-range connection of the amygdala to the septal and hypothalamic regions via the stria terminalis, in which the bed nucleus (BED) is one of the primary destinations. The approximate locations of the four panels in Figure 4 are shown in Figure 1. In Figure 4A, three distinctive white matter tracts of the limbic system—stria terminalis (st), fimbria (fi) and alveus (alv)—were well resolved, showing the stria terminalis anatomically separated by the lateral ventricle, even though its spatial location and trajectory were similar to those of the fimbria. In Figures 4B,C, the fimbria was connected to the fornix, and the stria terminalis remained in close proximity to the fimbria/fornix (see also Figure 1). Throughout the course of its trajectory, the stria terminalis (poorly myelinated white matter) had a lower FA value (and is therefore darker on the color-coded map) and longer T2 (therefore brighter on the b0 image) than the fimbria/fornix. In Figure 4D, the stria terminalis is about to complete the connection to the bed nucleus (BED), and the white matter bundle containing the amygdalofugal pathway (asterisk) can be seen projecting toward the hypothalamus.

### Interpretation of Conventional *In Vivo* DTI Based on Mesoscale Data

**Figure 5:**
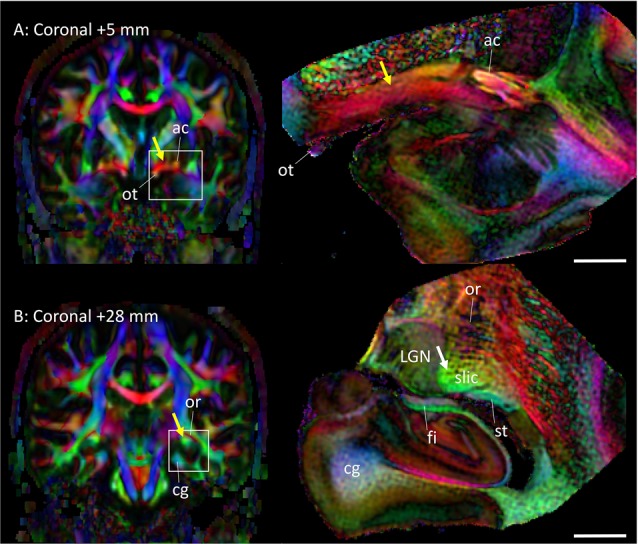
**Assignments of two anatomical regions that were not well resolved in previous DTI studies.** Left column: *in vivo* DTI images. Right column: postmortem DTI images at corresponding slice levels. **(A)** Yellow arrow, area known as the substantia innominata, which contains gray matter structures (e.g., basal nucleus and lateral hypothalamus) and white matter tracts (e.g., ansa lenticularis, ansa peduncularis, amygdalofugal pathway, sublenticular stria). Some of these structures are visible on the postmortem mesoscale DTI (Figures 2, 3), but the structures are diffuse and the boundaries are often vague. **(B)** Yellow arrow, high anisotropy area that can be identified reproducibly by *in vivo* DTI. The mesoscale DTI resolved this area into the fimbria, stria terminalis, and another white matter structure tentatively assigned to the sublenticular part of the internal capsule (white arrow). Abbreviations: ac, anterior commissure; cg, cingulum; fi, fimbria; LGN, lateral geniculate nucleus; or, optic radiation; ot, optic tract; slic, sublenticular part of the internal capsule; st, stria terminalis.

Figure 5 highlights two areas where contrast interpretation and anatomical assignment had not been clearly made in the past from *in vivo* DTI images. Figure 5A highlights an area of the basal forebrain sometimes called the “substantia innominata.” As this area consists of diffuse gray matter nuclei and several white matter tracts, clear anatomical assignment had been difficult, even with histological analysis. As also detailed in Figures 2C,D, the gray matter nuclei include the basal nucleus of Meynert (BN) and the lateral hypothalamus; these nuclei interdigitated with various axonal fibers, including the ansa lenticularis (al), ansa peduncularis, sublenticular stria (sls) and the amygdalofugal pathways, some of which could be identified only in limited areas where they had relatively compacted forms. This area had been reproducibly recognized in previous *in vivo* DTI data, with a very strong structural alignment with the medial-lateral (red) orientation and intermediate FA values (0.4–0.6). In a recent study using *in* *vivo* DTI (Kamali et al., 2016) suggested that this area represented the amygdalofugal pathway. Our results confirm that claim, but it should be noted that the amygdalofugal pathway was only one of the constituents of this area, as annotated in the panels in Figure 2. On T2-weighted images (Figures 2C,D), this area had an intensity signature similar to that of the gray matter—brighter than nearby white matter, such as the anterior commissure (ac) and the fronto-occipital fasciculus (ifo). As previous anatomical descriptions have explained, this is an area with both gray and white matter characteristics. An investigation of this area with DTI could be an interesting research target, but careful interpretation would be required.

Figure 5B shows an area related to the stria terminalis, where we typically find a white matter region with high FA (>0.6). This area was designated as the stria terminalis/fornix in previous atlases (Wakana et al., 2004; Mori et al., 2008; Oishi et al., 2011). The mesoscale DTI revealed that three major tracts are clustered there, including the stria terminalis and fornix, while the assignment of the third tract with high FA, indicated by the white arrow is, according to Mai et al. (2016), the sublenticular part of the internal capsule (slic). One interesting observation was that this white matter structure (slic) maintained a spatially close relationship with the amygdala (Figures 2E–I) and could contain projections from/to the temporal lobe and brainstem, as previously suggested by Rafal et al. (2015). In any case, the interpretation of *in vivo* DTI data in this area requires caution because of the mixture of three tracts with very different trajectories.

### Anatomical Variability

**Figure 6:**
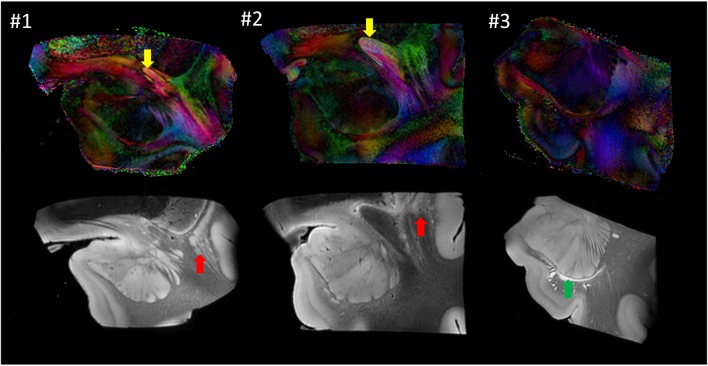
**Comparison of the three postmortem samples as color-coded orientation maps and b0 (T2-weighted) images.** Sample #1 was used for histology. Arrows: anterior commissure (red); claustrum (yellow); lateral ventricle (green).

Figure 6 shows images from each sample at similar anatomical locations. Although preliminary, with only three samples, the postmortem images revealed a considerable amount of anatomical variability at the mesoscale level. For example, the location of the anterior commissure, which is one of the most heavily used anatomical landmarks by which to judge the image registration, was well matched between #1 and #2 (yellow arrows), but the sizes were considerably different. This amount of variability of the anterior commissure can also be found in different sources of human brain histology data (Nieuwenhuys et al., 1988; Nolte and Angevine, 2000; Mai et al., 2008). Between these two samples, the anatomy of the surrounding gray matter structures was also variable. For example, a large portion of the ventral section of the claustrum was clearly visible in #1 (red arrow) but not in #2. In #3, the temporal horn of the lateral ventricle was visible (green arrow), whereas it was closed and not identifiable in samples #1 and #2.

## Discussion

### The Role of High-Resolution Data from MRI and Histology in Postmortem Samples

We used high-resolution postmortem DTI to elucidate the structure of the white matter tracts related to the amygdala, which was validated by subsequent serial histology examinations of these tracts. Our results show the potential of combining these complementary imaging modalities at different scales to elucidate neuroanatomy in detail. Histology of tissue sections can delineate anatomical structures at the sub-micron level. However, the orientation of axonal structures is difficult to quantify using conventional histology. Recently, polarized light imaging has revealed white matter architecture in remarkable detail, at the single-axon level (Axer and Keyserlingk, 2000; Axer et al., 2016; Zeineh et al., 2016); but such observations remain mostly 2D, and extension to 3D analysis would require cellular-level precision of registration through tens of consecutive histological sections. Mesoscale (100–500 μm) imaging methods, therefore, provide an interesting opportunity that complements histology-based and *in vivo* MRI-based observations by which to delineate white matter architecture.

Our knowledge about human brain anatomy remains surprisingly limited. Many important circuits, such as the extrapyramidal motor system, visual pathways and hippocampal–entorhinal connections, have been studied extensively using animal models, but their equivalents in the human brain are not always clear, and are often depicted as conceptual diagrams and cartoons. Although manual dissections of the human brain, such as those by Krieg (1963), are widely referenced as the gold standard of macroscopic tract anatomy, this approach requires a substantial amount of subjective judgment during dissection. The combination of postmortem high-resolution MRI and co-registered histology data could be an important approach to deepen our understanding of human brain anatomy. We want to stress that the amygdala has many more connection pathways beyond the stria terminalis and the amygdalofugal pathway. These include connections to various cortical areas and the brainstem; although confirmed in animal studies, these connections have not been identified as discrete and specific tracts in histology-based anatomical descriptions. It is possible to explore such connections using high-resolution DTI and tractography, but a careful approach is needed because it will be difficult to validate such results without sound histology.

Another interesting observation in this study was the intricate intra-amygdalar laminar and white matter structures delineated by b0 (T2-weighted) and color-coded images (Figure 2). Using the 3D tract reconstruction approach, their structural continuity with the amygdalofugal pathway was also delineated (Figure 3). As these intra-amygdalar structures have not been extensively studied in the human brain to date, it would be of great interest to extend this integrated MRI–histology analysis to further elucidate their anatomy and interactions with various amygdalar nuclei. Such studies, however, would require a careful and comprehensive approach with a much larger sample size.

### Applications of the Present Findings to *In Vivo* MRI Studies

In recent years, the connectivity of the amygdala has been extensively studied in various disease models using diffusion MRI. A simple PubMed search using “diffusion,” “MRI” and “amygdala” returned more than 200 publications, half of which were within the past 3 years. The high resolution (250 μm) used in the present study should bridge the gap in knowledge gained from histology and accumulating *in vivo* MRI data, and provide new insight into how the amygdalar white matter structures are revealed in MRI data.

One of the most limiting factors of diffusion MRI is the spatial resolution, which is limited to 1.5–2.5 mm within a practical scanning time. Figures 4, 5 have 5-mm scale bars allowing the determination of which anatomical structures can be reliably investigated from a given voxel size (for example, 2 mm). In many areas, the fornix occupies one or two voxels and the stria terminalis occupies one voxel. In addition, the stria terminalis has a lower FA value than typical white matter tracts. Although the stria terminalis has been delineated previously using DTI (Wakana et al., 2004; Mori et al., 2008; Oishi et al., 2011; Kamali et al., 2015), it could be challenging to reproducibly delineate its detailed anatomy with *in vivo* DTI.

In Figures 2–5, it is clear that even major white matter bundles, such as the cerebral peduncle (cp), internal capsule (ic) and optic radiation (or), have highly complex microscopic structures. It is well known that DTI with a 2-mm voxel size contains many white matter tracts with different orientations and connections, which are clearly delineated in these figures. To retrieve intra-voxel anatomical information, many advanced diffusion-based analyses have been postulated, which do not resort to tensor-based contraction of observations into six parameters per voxel (e.g., Frank, 2001; Tournier et al., 2002, 2004; Tuch et al., 2003). The limitations of these tools, however, are also known—they cannot clearly differentiate two crossing fibers with acute angles; the curves within a voxel are difficult to characterize; and complex tract configurations, such as crossing, kissing and funning, could complicate the analyses. For the validation of these tools, our mesoscale data, such as the anatomy of the stria terminalis, could serve as a test bed.

### Applications to Population-Based Analysis

The primary aim of the present study was to integrate observations of the amygdalar white matter structures from three different resolution scales; *in vivo* data (~1 mm), *ex vivo* data (~0.2 mm) and serial histology sections (~0.001 mm). Although our efforts focused on the cross-resolution integration of anatomical data, an interesting extension of this study would be to investigate cross-population variability and establish population-based coordinate systems, which will provide opportunities to perform quantitative anatomical analyses and potentially provide a population-based stereotaxic frame of the brain anatomy at mesoscale accuracy. Amunts et al. (2005) used histological sections to define multiple amygdalar nuclei from 10 postmortem samples, which were then registered to the 1-mm MNI coordinate system defined by MRI, generating a probabilistic atlas. This study, however, did not contain information about amygdalar white matter anatomy; and, importantly, the study was designed to enrich the anatomical information of atlases at the 1-mm scale.

Our preliminary data with three samples posed significant challenges in the creation of population-based atlases at the meso- and microscopic scales. This is partly due to the labor involved in the integrative MRI–histology studies (cross-population studies typically require a sample size of 20–30), and more importantly due to the degree of anatomical variability delineated in such high-resolution domains (highlighted in Figure 6). Namely, the amount of cross-population anatomical variability, which is in the order of a few mm, far exceeds the image resolution at the 250 μm (MRI) or 1 μm (histology) scales. When *in vivo* MRI data, such as T1-weighted images, are transformed for cross-subject registration, structures that look homogeneous, such as the white matter, are treated as freely deformable media to align T1-identifiable structures (e.g., various gray matter nuclei and the cortex); the deformation of areas with homogeneous intensity has no impact on cost functions or on visual inspection of the registration quality. The cross-subject alignment of the mesoscale MRI or histology data is much more complicated due to the lack of such freely deformable areas. The closure and emergence of ventricular spaces (Figure 6) would also pose a challenge to the alignment of structures at the mesoscale. Without manual guidance based on anatomical knowledge (such as that of the invisible “closed” ventricle spaces), automated registration algorithms would force alignment of ventricle shapes with markedly different anatomical configurations. As the scale of anatomical observation becomes finer, the concept of cross-subject anatomical registration itself becomes questionable. This is certainly an important topic that needs further attention (Qiu et al., 2008).

### Conclusion

This article describes a unique anatomical study integrating postmortem high-resolution MRI (mesoscale) and histology (microscopic) observations of white matter tracts associated with the human amygdala. The detailed anatomy of the two major innervating tracts, the stria terminalis and amygdalofugal pathway, were delineated at 250 μm resolution, and anatomical assignments were confirmed by subsequent histology of the same sample. This study should serve as a useful anatomical reference to guide the anatomical interpretation of future MRI studies.

## Author Contributions

SM: overall design, sample preparation, data analysis, manuscript. YK: overall design, sample harvest, sample preparation, histology, figures. ZH: sample preparation, imaging, data analysis. MA: imaging technology and protocol, data analysis. JP: data analysis, histology data processing. TB: histology data processing. MIM: data analysis technology, manuscript. DW: image technology and protocol, manuscript. JCT: sample harvest, sample preparation, histology, manuscript.

## Funding

This publication was made possible by the following grants by National Institute of Health: R01NS084957, EB015909, R01NS086888, NIH P50AG005146, and the Brightfocus Foundation.

## Conflict of Interest Statement

SM and MIM own “AnatomyWorks.” SM is its CEO. This arrangement is being managed by the Johns Hopkins University in accordance with its conflict of interest policies. The other authors declare that the research was conducted in the absence of any commercial or financial relationships that could be construed as a potential conflict of interest.

## Footnotes

^1^brainknowledge.mricloud.org
